# Development of Hematopoietic and Endothelial Cells from Human Embryonic Stem Cells: Lessons from the Studies using Mouse as a Model

**DOI:** 10.1100/tsw.2007.310

**Published:** 2007-12-10

**Authors:** Anna Jezierski, Albert Swedani, Lisheng Wang

**Affiliations:** ^1^ Department of Biochemistry, Microbiology and Immunology, University of Ottawa, Ottawa, ON, Canada; ^2^ Stem Cell Biology Group, Ottawa Health Research Institute, University of Ottawa, Ottawa, ON, Canada; ^3^ Ottawa Institute of Systems Biology, University of Ottawa, Ottawa, ON, Canada

**Keywords:** human embryonic stem cells, hematopoiesis, hemangioblast, endothelial precursors

## Abstract

The current progress using the human embryonic stem cell (hESC) model system has provided much insight into the early origins of the hematopoietic and endothelial lineages, particularly the elusive hemangioblast. Recently, the cellular hierarchy and molecular regulation controlling hematopoietic commitment have been further elucidated. These findings not only provide new insights into early human development, but also advance the knowledge required to develop techniques capable of generating a given cell type for potential clinical applications. This review will focus on the latest advances using the hESC model system, capitalizing on the well-established mouse embryonic stem cell model system, as a means to investigate the lineage commitment events underlying the early embryonic development of human hematopoietic and endothelial cells.

## BACKGROUND

Current knowledge of hematopoiesis (from the Greek hemato meaning "blood" and poiesis meaning "to make") and endothelial cell (i.e., cells lining the inside of blood vessels) development in the mammalian embryo is largely based on studies of the mouse system[1,2,3]. In general, during mammalian embryogenesis, hematopoiesis occurs transiently at a number of successive anatomical locations[4]. Primitive hematopoiesis occurs within the blood islands of the extraembryonic yolk sac. This early hematopoietic commitment is observed through the generation of primarily nucleated erythrocytes that express embryonic and fetal hemoglobins, and later some myeloid lineage and primitive macrophage cells[5]. These primitive erythroblasts have been shown to enucleate in circulation[6] and eventually switch towards a more mature (adult) hemoglobin expression pattern[7]. Simultaneously, endothelial cells have also been shown to emerge in the blood islands leading to concurrent vasculogenesis[8], which suggests that both lineages may be derived from a putative common precursor termed the hemangioblast.

Definitive hematopoietic stem cells (HSC), which have self-renewal capacity and sustain blood cells throughout the lifetime of an individual, emerge independently within the intraembryonic aorta-gonad-mesonophros (AGM) region[9]. These cells then colonize the fetal liver and give rise to all other hematopoietic lineages, including myeloid (red and white blood cells, platelets) and lymphoid lineages (T and B lymphocytes, plasma cells). Although the developing fetal liver serves as the main hematopoietic organ of the fetus, the precise relative contribution of the AGM and yolk sac hematopoietic precursors to the developing HSC pool remains unclear[4]. Recent work, employing an *in vivo* cell tracing technique, has shown that hematopoietic precursors from the yolk sac migrate to the fetal liver and thymus, and that yolk sac blood islands contain precursors of adult HSC[10]. The placenta may also play a role in providing a microenvironment for developing HSCs, although its role in the generation of HSCs remains unknown[11]. Following birth, the bone marrow serves as the principal hematopoietic organ into adulthood. It sustains the life-long maintenance of HSCs and ensures continual provision of specialized mature blood cells.

Despite the well-characterized events underlying early hematopoiesis in the mouse, a clear understanding of human hematopoietic and endothelial development is lacking. The scarcity and ethical complications of using early-stage human embryos, as well as the narrow time frame during which hematopoietic and endothelial development is initiated, have significantly hindered our understanding of these early embryonic events. The emergence of human embryonic stem cells (hESCs) provides a unique model system to study the earliest events of human development that are otherwise difficult to access *in vivo*.

## EMBRYONIC STEM CELLS: A POWERFUL TOOL TO RECAPITULATE *IN VIVO* HEMATOPOIETIC AND ENDOTHELIAL DEVELOPMENT

### Mouse ESCs Pave the Way for hESCs in the Study of Hematopoietic and Endothelial Development

Prior to the establishment of hESC lines (1998), studies using mouse ESCs (mESCs) produced a wealth of knowledge regarding early developmental decisions pertaining to embryonic hematopoietic and endothelial development *in vitro*. Keller and colleagues pioneered the use of differentiating mouse embryoid bodies (mEBs, formed by cellular aggregation of mESCs) as a tool to study early hematopoiesis. Of significance has been the capacity of mESCs to differentiate into hematoendothelial precursors, which in turn, recapitulate *in vivo* mouse embryonic hematopoiesis[12,13,14,15]. Moreover, the temporal appearance of hematopoietic and endothelial precursors, in addition to the differential expression pattern of mesodermal, hematopoietic, and endothelial genes in developing mEBs, have been well characterized and shown to imitate normal embryogenesis[16,17,18,19,20] (reviewed by Keller[21]).

Employing mESCs to the study of hematopoietic and endothelial specification *in vitro* paved the way for the use of hESC as a means to elucidate the human hematopoietic and endothelial development. hESCs are derived from the inner cell mass of blastocyst-stage embryos[22]. These cells, which are capable of self-renewal and long-term propagation, maintain karyotypic integrity in culture and possess the developmental potential to differentiate into derivatives of all three embryonic germ layers: endoderm, ectoderm, and mesoderm[22]; hence, giving rise to almost any tissue of the body. Of these layers, the mesoderm is capable of further development into muscle, skeleton, and endothelial and hematopoietic cells. Under appropriate culture conditions, by either cellular aggregation (hEB formation)[23,24,25,26, 27,28,29] or coculture with stromal cells[30,31], hESCs have the ability to differentiate into multiple hematopoietic lineages[23,24,25,26,30,31], as well as endothelial cells[25,27,28,29,32]. *In vivo* transplantation results suggest that hESCs may also give rise to hematopoietic stem/progenitor cells[33,34,35]. Based on these biological characteristics, hematopoietic and endothelial differentiation of hESCs recapitulates many aspects of embryonic development and provides an *in vitro* model[21] for studies that could not otherwise be well examined in the human embryo. The ability to decipher the mechanisms controlling germ layer specification, cellular commitment, lineage restriction, and terminal differentiation of hESCs will eventually allow scientists to direct the differentiation of hESCs into specific cell types for cell replacement therapy.

## Similarities and Differences between Mouse and Human ESCs

Although there is a great deal of similarity between the technical aspects of mouse and human ESC maintenance and differentiation, it is important to note that the knowledge derived from one species cannot be arbitrarily applied to another. Fundamental differences exist between mouse and human developmental hematopoiesis. Most notably, humans contain an anatomically more complex primary and secondary yolk sac in addition to a shortened yolk sac phase[36] and earlier onset of medullary hematopoiesis[37].

In addition, established maintenance and differentiation strategies for mESCs differ significantly from those for hESCs (summarized in Table 1). For example, basic fibroblast growth factor (bFGF), an essential growth factor for the maintenance of hESCs[38,39,40,41], stimulates mESC differentiation into neural tissues[42]. Bone morphogenetic protein 4 (BMP4), an essential factor for mESC self-renewal[43], induces hESC differentiation[41,44]. Activin A (a member of the transforming growth factor-β [TGF-β] family) maintains hESCs, but not mESCs, by regulating bFGF and BMP4 pathways[45,46,47,48].

**TABLE 1**
**Differences between Undifferentiated Human and Mouse ESCs**

| Proteins and Functions | Human ESCs | Mouse ESCs |
|---|---|---|
| Leukemia inhibitory factor (LIF) receptor | Absence | Presence |
| LIF function | No effect | Maintenance |
| Bone morphogenetic protein 4 (BMP4) | Differentiation | Maintenance |
| Basic fibroblast growth factor (bFGF) | Maintenance | Differentiation |
| Transformation growth factor (TGF-β) | Maintenance | Differentiation |
| Activin A | Maintenance | Differentiation |
| SSEA-1 | Absence | Presence |
| SSEA-3 | Presence | Absence |
| SSEA-4 | Presence | Absence |
| Flk1/KDR | Presence (~15% of hESCs) | Absence |

* Reference: [22,25,26,41,43,47,48,57,111,112,113,114,115,116,117].

Furthermore, hESCs are also insensitive to leukemia inhibitory factor (LIF), a growth factor essential for the maintenance of mESCs[22,49]. LIF and related cytokines function by activating signal transducer and activator of transcription (STAT3) via the gp130 receptor[50]. BMP4 blocks differentiation by inducing the expression of inhibitor of DNA binding/differentiation (Id) proteins, which enables mESC self-renewal in response to LIF/STAT3[43]. In contrast to mESCs, cellular interplay and paracrine signaling may be required for the self-renewal and pluripotency of hESCs[51]. For example, inhibition of bFGF and TGF-β, which instruct hESC differentiation, is balanced by self-renewal signals controlled by insulin growth factor (IGF)[51]. IGF seems to be produced in response to bFGF induction by hESC-derived fibroblast cells, which provide supportive factors that sustain the defining properties of hESCs[51]. In addition to the aforementioned differences, certain cell surface markers and transcriptional factors are also differentially expressed in mESCs and hESCs, such as stage-specific embryonic antigen

(SSEA) -1, -3, and -4. SSEA-1 is exclusively expressed in mESCs, whereas, SSEA-3 and -4 are exclusively expressed in hESCs.

Such differences between mouse and human ESCs may reflect real differences between species or the precise embryonic origin of both cell types. Currently derived pluripotent mouse EpiSCs (postimplantation epiblast-derived stem cells that generate the embryo proper) share patterns of gene expression and signaling responses with hESCs[47,48]. The Activin/Nodal pathway that was important in the maintenance of hESCs is also crucial for the self-renewal of the mouse EpiSCs[47,48]. Hence, further study and comparison of EpiSCs and hESCs may contribute to the understanding of early human development.

Despite the differences between mouse and human ESCs, different hESC lines manifest a remarkable degree of phenotypic similarity and harbor a common set of markers, as recently characterized by the International Stem Cell Initiative[52]. This comparative study covered 59 different hESC lines maintained in 17 laboratories scattered across 11 countries, the largest cross-sectional analysis to date. Despite unique genetic backgrounds, derivation and maintenance techniques under varying culture and passage conditions, the 59 hESC lines examined revealed a great deal of similarity. The degree of phenotypic convergence within hESC lines worldwide is reassuring and substantiates the use of hESCs as a powerful tool to study human hematopoiesis and endothelial specification.

## SURROGATE MARKERS USED IN THE STUDY OF HEMATOPOIETIC AND ENDOTHELIAL DEVELOPMENT

In addition to functional assays, surrogate markers, such as cell surface proteins and gene expression profiles, have been used to characterize the successive development of hematopoietic and endothelial precursors, and subsequently committed cell lineages. These surrogate markers can be analyzed by flow cytometry, PCR, and imaging methods.

Commonly, early human endothelial and hematopoietic precursors have been characterized based on the expression of KDR (mouse counterpart Flk-1, also known as vascular endothelial growth factor receptor 2), CD34 and CD31 (platelet endothelial cell-adhesion molecule-1, PECAM-1), together with the absence of pan-leukocyte marker CD45[25,26,27,29,37,53,54]. Recently, leukosialin (CD43) has been proposed as an alternative marker for hESC-derived hematopoietic stem/progenitor cells[55]. Following hESC differentiation, Vodyanik et al. found that $CD34^{+}CD43^{+}$-derived cells were hematopoietic cells, $CD34^{+}CD43^{-}CD31^{+}KDR^{+}$ cells were endothelial cells, and $CD34^{+}CD43^{-}CD31^{-}KDR^{-}$ cells were mesenchymal cells[55].

Multiple markers have been used for the characterization of endothelial precursors and mature cells. Expression of vascular endothelial cadherin (VE-cadherin, CD144) alone[56,57] or together with the ability to take up Dil-labeled acetylated low-density lipoprotein (Dil-Ac-LDL)[56,58] has been used as a marker for the identification of endothelial precursors. CD31, CD34, Flk-1, and von Willebrand factor (vWF) are generally used for the identification of endothelial precursor and/or mature endothelial cells[57,58,59]. One of the commonly used markers for discriminating committed endothelial from hematopoietic cells is VE-cadherin[59]. Committed nucleated hematopoietic cells express CD45, but not VE-cadherin. In addition to the cell surface proteins, a large number of signaling molecules and transcription factors are involved in hematopoietic and endothelial development (such as KDR/Flk-1, c-kit, Scl/Tal1, Tie2, Gata2, and Runx1, etc.) (reviewed[21,60]).

## COMMON PRECURSOR HYPOTHESIS: THE HEMANGIOBLAST

A central theme surrounding the emergence of the hematopoietic system is the existence of a common bipotent precursor with endothelial properties. Endothelial and hematopoietic cells, in the developing vertebrate embryo, emerge in close spatial and temporal association in the extraembryonic yolk sac blood islands at the 3rd week of gestation in the human embryo and at embryonic day 7.5 in the mouse embryo[53,61]. These blood islands consist of aggregates of mesodermal cells that contribute to both the vascular and hematopoietic system. The cells at the periphery develop into endothelial cells, whereas the inner cells develop into hematopoietic cells[62]. This close developmental proximity of both lineages, initially observed in the avian embryo[63], led to the proposition of a common mesodermal precursor, termed the hemangioblast, capable of giving rise to both endothelial and hematopoietic cells[63,64]. Indeed, more recent mapping studies in the developing mouse embryo reveal that the hemangioblast arises as a transient mesodermal population within the posterior region of the primitive streak[20].

The hemangioblast was initially identified using the mESC differentiation model[13,16,57] and later *in vivo* in the mouse embryo[20], in zebrafish[65], and recently in the hESC model[25,26,27]. Although the precise origin of hematopoietic and endothelial lineages remains a topic of ongoing debate, the concept that endothelial and hematopoietic cells share a common bipotent precursor has been intensely pursued (reviewed[66,67,68]). In addition to contributing to the understanding of early embryonic development, the existence of the hemangioblast is particularly promising as an alternative source of hematopoietic and endothelial cells for cell replacement therapies.

## PROPOSED DEVELOPMENTAL HIERARCHY OF ENDOTHELIAL AND HEMATOPOIETIC CELLS: THE MOUSE MODEL

Studies in the mouse have suggested that both yolk sac and AGM-derived precursors are the progeny of a bipotent hemangioblast (reviewed[21]). Evidence supporting this common origin of endothelial and hematopoietic cells arose through the identification of a blast colony-forming cell (BL-CFC) derived from mEBs and later the mouse embryo[13,16,20,57].

BL-CFCs have the potential to differentiate along both lineages in response to VEGF (vascular endothelial growth factor, a ligand for the receptor tyrosine kinase, Flk-1/KDR)[69,70] prior to the onset of primitive erythropoiesis[13,16,20]. These VEGF-responsive precursors develop within the mammalian yolk sac blood islands as a subset of mesoderm (Brachyury$^+$) cells expressing Flk-1[17,20,71]. BL-CFCs give rise to primitive and definitive hematopoietic as well as endothelial precursors[13,16,57,71,72] (Fig. 1). Targeted deletion of Flk-1 in mESCs dramatically disrupted blood vessel formation and failed to contribute to primitive and definitive hematopoiesis *in vivo*[73,74]. Further, *in vitro* and *in vivo* studies have shown that Flk-1 is required for the migration and expansion of the hemangioblast[73,74,75,76] from the primitive streak to the yolk sac, a site permissive for hematopoiesis[73,74,75,76,77].

In addition to hematopoietic and endothelial cells, the BL-CFC also has the potential to give rise to other lineages under different culture conditions, such as smooth muscle cells[75]. Cell-lineage tracing experiments in mice have further demonstrated that cardiac lineages, endocardium, and myocardium develop from an intermediate cell population that expresses Flk-1[78,79,80]. Myocardial and endothelial lineages, as well as cardiomyocytes, have also been shown to arise from Flk-1$^+$ precursors isolated from differentiated mouse ESC and early embryos[78,81]. These Flk-1$^+$ precursors of cardiac potential, termed cardiovascular colony-forming cells (CV-CFCs), might arise from an intermediate Flk-1$^+$ cell population that emerges later and is distinct from the Flk-1$^+$ population with hemangioblastic properties[19]. The finding that CV-CFC precursors contain both cardiac and vascular potential[19] sustains the belief that cardiac lineages develop from a precursor with endothelial potential[82,83]. Hence, it appears that the BL-CFC, which gives rise to both hematopoietic and vascular lineages of the blood islands, precedes the development of the CV-CFC that generates the myocardial and endothelial lineages [19]. Complementary studies have shown that Flk-1$^+$CD31$^+$VE-cadherin$^-$ cells could act as cardiohemangioblasts to form cardiac cells[84], which is consistent with the findings that VEGF-induced cardiomyocyte differentiation of mESCs occurs via the activation of Flk-1 and Flt1 (vascular endothelial growth factor receptor 1)[85]. Collectively, these results suggest that Flk-1$^+$ cells may represent a multipotent mesodermal precursor that later specializes into different mesodermal lineages according to the position in the embryo.

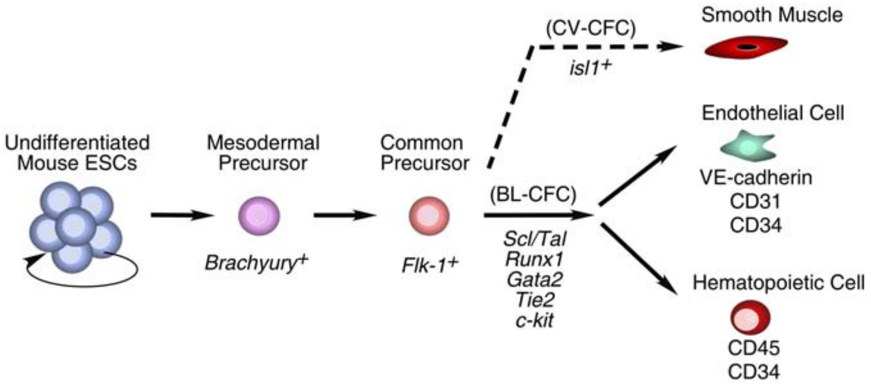

**FIGURE 1.** Proposed mouse developmental hierarchy of mesodermal cells**.** A subset of Brachyury positive cells, expressing Flk-1, emerges during mesodermal specification of differentiating mESCs. These Flk-1$^+$ cells are capable of giving rise to BL-CFCs, which can further develop into hematopoietic and endothelial cells, as well as smooth muscle cells. Consequently, the BL-CFC has been proposed to represent an *in vitro* equivalent of the hemangioblast. Furthermore, the same subset of Flk-1$^+$ cells can also give rise to CV-CFCs, which are endowed with cardiac and vascular potential. These CV-CFCs emerge later and are distinct from the BL-CFCs. Collectively, the BL- and CV-CFC may represent a transient precursor population, capable of commitment to the hematopoietic, endothelial, smooth muscle, and cardiac lineages dependent on the temporal developmental expression and activation of different transcription factors (such as Scl/Tal1, Runx1, Gata2, Tie2, c-kit, and isl, respectively). Signaling molecules and transcription factors are indicated in *italics*, whereas cell surface proteins are indicated in plain text.

Recently, Moretti et al. defined a mESC-derived multipotent Isl1$^+$ (LIM-homeobox transcription factor islet-1) cardiovascular progenitor cell expressing Flk-1 capable of generating cardiac muscle, smooth muscle, and endothelial cells *in vitro*[86]. Endothelial cells were found to arise solely from Isl1$^+$Flk-1$^+$ cells, representing a subset of more restricted progenitor cells, capable of generating endothelial and smooth muscle cells[86]. This result is in accordance with the observation that mESC-derived Flk-1$^+$ cells can differentiate *in vitro* and *in vivo* into two major components of the vascular system: endothelial and mural cells[87]. These Flk-1$^+$ cells expressing PDGFR (platelet-derived growth factor receptor) -β have the potential to differentiate into mural cells in the presence of PDGF[87,88]. Rolny et al. found that the expression of PDGFR-β on CD31$^+$CD41$^+$Flk-1$^+$ early hematopoietic/ endothelial precursors regulates the vascular/hematopoietic development by affecting the differentiation rates of both endothelial and hematopoietic lineages[89]. In differentiating mESCs and yolk sac, activation of PDGFR-β was found to promote endothelial, but suppress hematopoietic, differentiation[89].

Altogether, increasing evidence suggests that Flk-1 expression marks a broad spectrum of mesodermal precursors. Although, conventionally, Flk-1 was regarded as a putative hemangioblastic marker, it also marks a common multipotent mesodermal precursor that can give rise to cell types like smooth muscle, cardiac, and mural cells. This further specialization of subsets of Flk-1 expressing cells might occur via the activation of other lineage-specific transcription factors during development.

## TRANSITION TOWARDS hESCS IN STUDYING THE HEMANGIOBLAST

Limited research with human embryos also suggests that human hematopoietic cells emerge in close vicinity to vascular endothelial cells during the early weeks of human gestation. Hematopoietic cell clusters were shown to arise from the AGM region between the 27$^{th}$ and 40$^{th}$ day of human development, through an intermediate blood-forming endothelium stage[53,61]. However, the cellular and molecular mechanisms associated with early human hematopoietic and endothelial genesis, prior to 3 weeks of gestation, remain elusive. The establishment of hESCs provides an alternative tool to tackle these questions.

A number of *in vitro* studies using hESCs also supports the hypothesis that hematopoietic and endothelial cells develop from a common precursor or from hemogenic (blood forming) endothelial cells[25,26,27,28,29,90,91,92]. Collectively, these studies have shown that: (1) the differentiation of hESCs into endothelial and hematopoietic cells follows a reproducible timing pattern[25,26,27, 28,29,30,32], (2) endothelial and/or hematopoietic cells develop from an intermediate precursor[25,26, 27,28,29], and that (3) these intermediate precursors are hemangioblasts[27] or primitive endothelial-like cells with hemangioblast properties[25,26] (see Fig. 2).

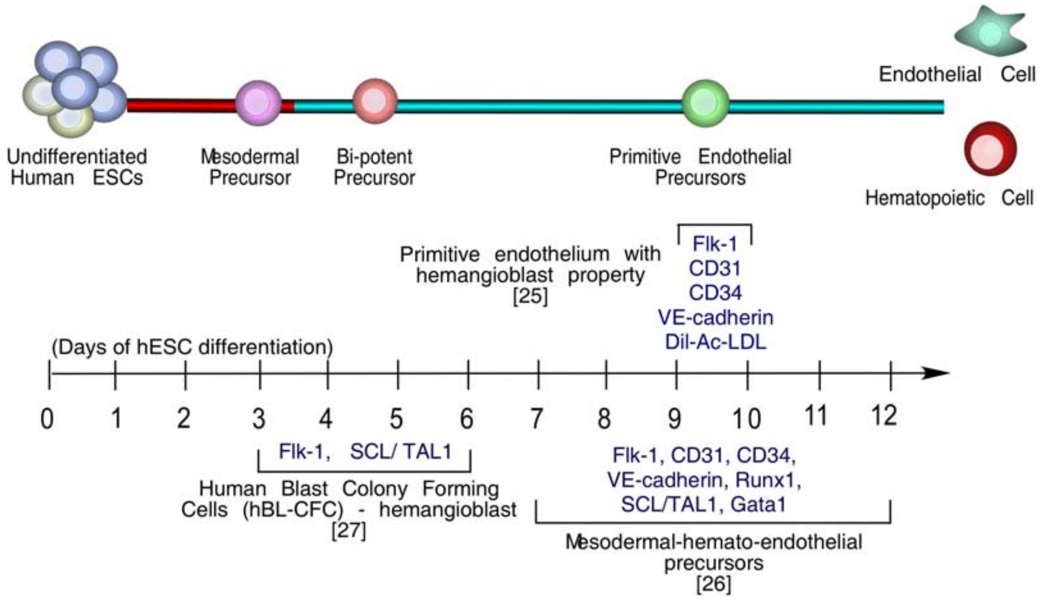

**FIGURE 2.** The schematic development of hESCs into hematopoietic and endothelial lineages. Two independent research groups have shown that hematopoietic and endothelial cells arise from an intermediate primitive endothelial-like or mesodermal hematoendothelial precursor with hemangioblast properties. Using an experimental approach similar to those employed in mESCs, the third research group recently demonstrated that a Flk-1$^{+}$ progenitor, derived from 3- to 4-day-old differentiated hEBs, is capable of giving rise to a transient human blast colony-forming cell (hBL-CFC). These hBL-CFCs have hemangioblast properties and are capable of further development into hematopoietic and endothelial lineages. Since various research groups employ different experimental approaches, it remains unclear whether the same or distinct populations of bipotent precursors were examined.

Hematopoietic differentiation from hESCs, via spontaneous formation of hEBs, mimics the early events of human yolk sac blood development, through mesodermal-hemangioblast differentiation followed by primitive erythromyeloid hematopoiesis[26,92]. Early-stage primitive endothelial precursors, derived from day 9 to 10 of hEB differentiation, coexpress endothelial markers PECAM-1, Flk-1 and VE-cadherin and are capable of Dil-Ac-LDL uptake, but lack CD45 expression[25]. This subpopulation of precursor cells, termed CD45$^{neg}$PFV, was capable of endothelial maturation as well as hematopoietic development when cultured under appropriate conditions and as a result, proposed to represent bipotent precursors with hemangioblast properties (reviewed[93]). Similarly, an equivalent cell population was derived following coculture of hESCs with OP9 stromal cells for 8 to 9 days[31], or with mouse bone marrow stromal cells (S17) or mouse yolk sac ECs (C166) for 17 days[30]. Using a different approach, Zambidis et al. showed that “mesodermal-hemato-endothelial colonies” derived from cells isolated between day 7 to 12 of hEB differentiation (expressing CD34, CD31 and Flk-1) were both competent for endothelial maturation and endowed with blood-forming potential[26]. These results suggest that hemangioblasts, with primitive endothelial properties, may exist during early hESC differentiation.

Despite these promising advances, a clonogenic bipotent human hemangioblast has remained elusive in the hESC culture. In the mouse developmental system, it is thought that the hemangioblast is likely contained within a subset of Flk-1$^+$ mesoderm cells that can be efficiently enriched for in developing mEBs[71]. The observation that Flk-1$^+$ cells emerge in early-somatic human embryos, at the beginning of blood vessel development, also suggests that Flk-1 plays a role in the regulation of human embryonic hematopoietic and vascular development[54]. In effect, Kennedy et al. recently identified Flk-1$^+$ precursors during early hEB differentiation capable of giving rise to blast colonies with both hematopoietic and endothelial potential[27]. These Flk-1$^+$ BL-CFCs represent a transient cell population that develops between day 3 and 4 of hEB differentiation, preceding the development of the primitive erythroid precursors[27]. Since CD31 was first detected on a subpopulation of Flk-1$^+$ cells at day 5, it may represent a distinct or earlier precursor population than the CD45$^{neg}$PFV precursors[25]. Two distinct populations of cells developed from the Flk-1$^+$ BL-CFCs. One population showed hematopoietic potential capable of further development into primitive erythroid, macrophage, and endothelial cells, while the other population gave rise to only primitive erythroid and endothelial cells[27]. These results may characterize the earliest stage of hematopoietic and endothelial commitment through an intermediate hemangioblast population. However, due to various research groups employing different experimental approaches[25,26,27], it is not clear whether the same or different populations of potential bipotent precursors were examined.

Although precursors with hemangioblastic properties (CD45$^{neg}$PFV cells and Flk-1$^+$ BL-CFC) have been demonstrated through the hEB differentiation system, large-scale generation or functional assessment of these putative hemangioblasts has not been achieved due to their rarity in culture. To circumvent this limitation, Lu et al. developed an *in vitro* differentiation method for generating large numbers of hemangioblasts from hESC-derived blast cells (hESC-BCs)[28]. These hESC-BCs developed from individual cells dissociated from early-stage hEBs (~3 days) and plated in serum-free semi-solid blast-colony growth medium for 3 to 6 days. These hESC-BCs expressed several proteins associated with the hemangioblast, but not CD31, CD34, and Flk-1; that were shown to be expressed on the hemangioblast population by other groups[25,26,27]. Nevertheless, the hESC-BC–derived single-cell suspension *in vitro* gave rise to colonies of erythrocytes, granulocytes, macrophage, megakaryocyte, and multilineage hematopoietic as well as endothelial cells. In order to determine endothelial functionality *in vivo*, fluorescently labeled hESC-BC were injected into mice with ischemia-reperfusion injury to the retina where they localized to the site of injury in the damaged vasculature and appeared to participate in repair[28]. Since the expression of FLK-1, CD31, and CD144 appeared following induction towards endothelial cell differentiation, these hESC-BCs represent a distinct and even earlier population of cells than those reported recently by Kennedy et al.[27].

In contrast to the commonly used hEB differentiation approach, Wang et al. recently developed a culture system to differentiate hESCs into hematopoietic and endothelial lineages by by-passing hEB formation altogether[29]. To initiate differentiation, hESCs were plated on mouse embryonic fibroblasts in differentiation media. Following exposure to a cocktail of growth factors, hESC-derived CD34$^+$ cells (of which a majority also coexpress CD31$^+$) purified following day 10 of differentiation were capable of giving rise to endothelial cells. These hESC-derived endothelial cells formed functional blood vessels *in vivo* for 150 days. Compared to earlier studies[32], these hESC-derived blood vessels integrated into the host vasculature and served as blood conduits. The hematopoietic potential of the derived hematopoietic cells was not further investigated in this study.

Although the concept of a hemangioblast is supported by the coexpression of a number of shared genes by both lineages, no unique surface marker currently exists that solely identifies this intermediate precursor. Recently, Zambidis et al. proposed that the BB9 monoclonal antibody[94], which was previously used to identify a subpopulation of human adult bone marrow–derived CD34$^+$ precursor cells[95], could be used to identify a population of putative hemangioblasts. The emergence of BB9 expression directly correlated with the development of a hemangioblastic BL-CFC, prior to CD34 expression, following 6 to 10 days of hEB differentiation. CD34 was subsequently up-regulated upon further differentiation[94]. Sorted BB9$^+$CD34$^+$CD45$^-$ or BB9$^+$CD43$^+$CD45$^-$ hEB cells formed blast cells that gave rise to definitive AGM-type precursors as well as definitive type NK and B lymphocytes following culture on OP9 stromal layers[94]. However, more studies are required in order to validate whether BB9 represents an unequivocal hemangioblast marker.

If a unique surface marker for the hemangioblast can be identified, the current discrepancies in the kinetics of the hEB differentiation system will be clarified. These discrepancies are largely a result of different conditions used for mesoderm induction and hematopoietic specification. While Wang et al.[25] and Zambidis et al.[26] differentiated hEBs in medium containing fetal bovine serum and a cocktail of cytokines, Kennedy et al.[27] used a procedure that consisted of a serum-free medium and a mixture of cytokines followed by expansion of the hEB-derived single cells in serum-containing semi-solid medium. The use of different mixtures of cytokines and batches of fetal bovine serum may alter the kinetics of hEB differentiation and, hence, contribute to the variation between the different research groups.

## HEMANGIOBLAST: A RARE EVENT OF A COMMON PRECURSOR?

During development, two main paradigms exist in defining the emergence of hematopoietic and endothelial lineages. First is the concept of the hemangioblast, while the second assumes that hematopoietic cells are derived from the primitive or hemogenic endothelium. The hemangioblast has been demonstrated in both mouse and human systems, although the mechanisms that regulate its development and differentiation are not well understood[16,17,20,25,27,57,76]. A major obstacle in characterizing the hemangioblast is its rare occurrence: an average of 33 hemangioblasts per mouse embryo[20], 0.33% (one in 300) in the mESC-derived precursors expressing Flk-1[96], 0.2% (one in 500)[25] or 0.4–1.4%[27] in the hESC-derived single precursors expressing Flk-1 or CD31. This scarcity of ESC-derived precursors may imply that not all hematopoietic and endothelial cells are developed from an intermediate hemangioblast population. In fact, recent studies in the mouse have shown that the yolk sac blood islands do not arise from a clonal bipotential precursor[97] and that endothelial precursors emerge from mesoderm prior to the development of the hemangioblast[98].

Furthermore, using multiple color tags to identify clonal precursors *in vivo,* as a means to delineate cell precursor-progeny relationships, Ueno and Weissman found that the blood islands within developing mouse embryos were polyclonal and not hemangioblastic in origin[97]. Although endothelial cells were found to be largely derived from Flk-1$^+$ precursors, following lineage-marking experiments, hematopoietic cells were not[97]. As such, it is probable that separate endothelial and hematopoietic precursors give rise to their respective cell fates, independent of an intermediate hemangioblastic stage and not necessarily concomitantly. Furuta et al. found that a considerable number of angioblasts could be detected 1 day prior to hematopoietic activity in early gastrulating mouse embryos[98]. Initial cell-lineage tracing of cells originating from the primitive streak to the yolk sac in mouse embryos also failed to reveal a common hematopoietic and endothelial precursor[99]. If this is indeed the case, then the close developmental association of the two lineages within the blood islands does not necessarily mean that they arise from a common precursor, but rather that the close association is needed for their further development and function[97]. The latter observation leads into the second developmental paradigm: the primitive or hemogenic endothelium, as originally proposed by Sabin[64]. A discrete subset of endothelial cells transiently exhibit blood forming potential during vertebrate development[53,61]. Although it remains unclear how great a potential the primitive or hemogenic endothelium might have, the environment sustained by functional endothelial cells during early development is essential for the correct differentiation of hematopoietic precursors.

Since conclusive evidence of an *in vivo* hemangioblast is dependent on direct lineage labeling in the intact embryo, these studies are unlikely to be carried out in the human embryo. Nevertheless, the emerging studies using hESCs lend support towards the hemangioblast theory[27,28], fueling the ongoing debate.

## DEVELOPMENT OF HEMATOPOIETIC STEM/PROGENITOR CELLS FROM hESCS

Although both mouse and human ESCs can be effectively differentiated into hematopoietic precursors *in vitro*, the ability to generate transplantable hematopoietic stem cells remains very challenging[100]. Commonly, HSCs are examined by reconstitution of lethally irradiated mouse bone marrow. A low-level and short-term hematopoietic reconstitution of lethally irradiated mouse hosts was reported over 10 years ago, suggesting that mouse ESCs may be capable of giving rise to HSCs[101,102,103]. A recent report from one group supported this observation and showed a robust engraftment (>70%) at 20 weeks of intra bone marrow transplantation of mESC-derived hematopoietic cells into mice[104]. It would be very interesting to see whether such robust engraftment could be reproduced and the appropriate culture conditions for generating mESC-derived HSC defined.

In the hESC setting, a few research groups have currently reported that hESC-derived hematopoietic cells showed hematopoietic stem cell properties after transplantation into immunodeficient mice or fetal sheep[33,34,35]. Although the proliferation and migration was low, transplantation of $CD45^{neg}$PFV-derived hematopoietic cells into immunodeficient mice after 8 weeks resulted in multilineage hematopoietic repopulation including myeloid, lymphoid, and erythroid lineages[33]. Similarly, Tian et al. found a low level of hESC-derived $CD45^{+}CD34^{+}$ hematopoietic cells in the primary recipient mice and very low levels of human engrafts in the secondary recipients, indicating that long-term repopulating cells (HSC) from differentiated hESCs may exist[34]. Using the fetal sheep model, Narayan et al. recently demonstrated that hESC-derived hematopoietic cells ($CD34^{+}/Lin^{-}$ or $CD34^{+}/CD38^{-}$ cells) are capable of engrafting primary recipients[35]. Secondary recipient engraftment was subsequently investigated and, similar to primary recipients, very low-levels of human engrafts were identified.

The limited capacity to engraft hESC-derived hematopoietic cells in animal transplantation models could be caused by an abnormal expression of gene profiles, such as an up-regulation of HoxB and a down-regulation of HoxA cluster genes[33]. Expression of HoxB4 enables *in vitro* differentiated mESCs to repopulate irradiated mice although with poor lymphoid reconstitution[105]. However, it is more likely that such limited success is due to the inadequacy of the *in vitro* microenvironmental niche, which characterizes hematopoietic development of hESCs. Further defining these niches will eventually allow researchers to generate desirable numbers of HSC for cell-based therapy.

## CONCLUDING REMARKS

Although much progress has been made in recent years on the study of early hematopoietic and endothelial differentiation, using both mouse and human ESCs, the underlying mechanisms responsible for specifying hematopoietic and endothelial cell fates remain to be fully deciphered. Understanding the mechanisms that govern these cell fate decisions will ultimately lead to developing techniques for the induction of hESC differentiation towards desired cell lineages. Ideally, producing HSCs and endothelial precursors from tailored patient-hESCs would circumvent the lack of transplantable histocompatible cells. The current research progress in epigenetic reprogramming has shed light on this possibility. Three independent groups have reproduced Takahashi and Yamanaka's findings[106], and successfully engineered embryonic stem-like cells from normal mouse skin fibroblast cells[107,108,109]. By introducing just four pluripotency-associated transcription factors (Oct3/4, Sox2 {known to be under strict epigenetic control during development[110]}, c-Myc and Klf4) into mouse skin fibroblasts, researchers could produce cell lines with the properties of ESCs[106,107,108,109]. If this method can be transferred to humans in the future, patient-specific hESC and, subsequently, differentiated HSCs and endothelial precursors could be derived without the use of donated eggs or embryos.

## ACKNOWLEDGMENTS

This work is supported by an operating grant from the Canadian Institutes of Health Research (CIHR) MOP-158235, a CIHR New Investigator Award MSH-166732, and a fund from J.P. Bickell Foundation to L.W. The authors would like to thank Drs. P. Roy Walker and Li LI for their critical reading of the manuscript.
